# Supplementary material for: Rapid Quantitative Detection of Live Escherichia coli Based on Chronoamperometry
Source: Biosensors (Basel). 2022 Oct 8;12(10):845. doi: 10.3390/bios12100845 (PMC9599875; doi:10.3390/bios12100845)
Supplement: Supplementary file 1 [file biosensors-12-00845-s001.zip › biosensors-1916146-supplementary.pdf]

Rapid Quantitative Detection of Live *Escherichia coli* Based on Chronoamperometry

Zhuosong Cao [1,2,†], Chenyu Li [2,†], Xiaobo Yang [2], Shang Wang [2], Xi Zhang [2], Chen Zhao [2], Bin Xue [2], Chao Gao [2,3], Hongrui Zhou [2], Yutong Yang [2], Zhiqiang Shen [2], Feilong Sun [1,*], Jingfeng Wang [2,*] and Zhigang Qiu [2,*]

[1] School of Environmental & Chemical Engineering, Xi'an Polytechnic University, Xi'an 710600, China

[2] Tianjin Institute of Environmental Medicine and Operational Medicine, Tianjin 300050, China

[3] School of Marine Science and Technology, Tianjin University, Tianjin 300072, China

* Correspondence: sunfeilong@xpu.edu.cn (F.S.); jingfengwang@hotmail.com (J.W.); zhigangqiu99@gmail.com (Z.Q.); Tel.: +86-13227072798 (F.S.); +86-2284655003 (J.W.); +86-2284655308 (Z.Q.); Fax: +86-2284655020 (Z.Q.)

[†] These authors have contributed equally to this work and share first authorship.

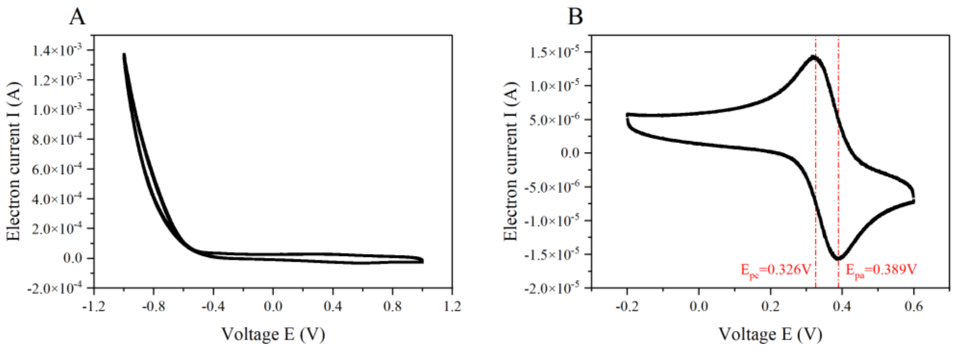

Figure S1. GCE activation process. A, activation cyclic voltammogram; B, detection cyclic voltammogram. After activation, ΔEp was <80 mV, and the peak current ratio was around 1:1, confirming that the GCE surface was free of impurities and that the reaction was sensitive.

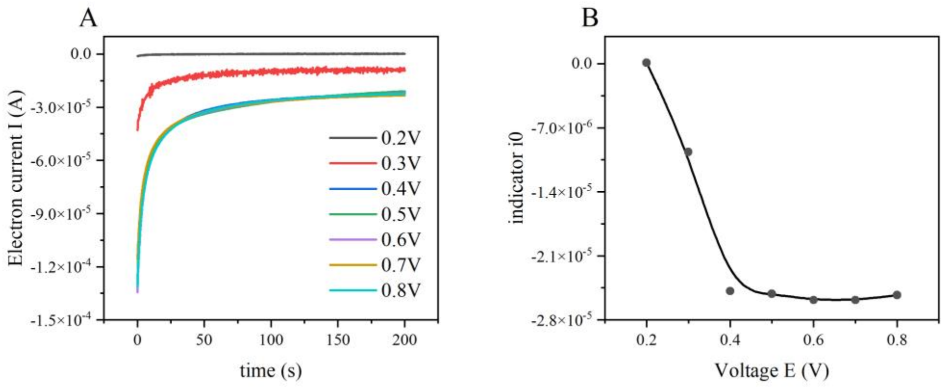

Figure S2. Step voltage selection. A, the response current under different step voltages; B, the stable current i_0 under different step voltages. When the step voltage was >0.5 V, the stable current i_0 did not change with the voltage, and the electrochemical process was controlled by diffusion. To ensure the stability of the electrochemical process, the step voltage used was 0.6 V.

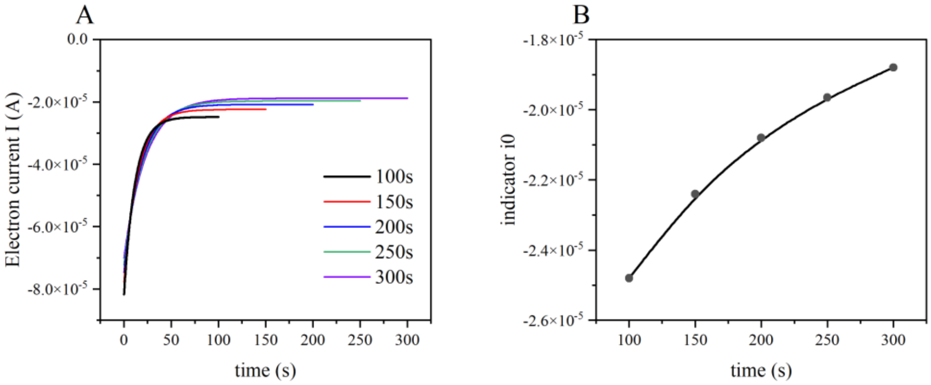

Figure S3. Detection time selection. A, the response current under different detection times; B, the stable current i_0 under different detection times. When the detection time reached 200 s, the current was basically stable. Excessively high detection time might lead to the accumulation of surface-adsorbed *E. coli*, affecting the detection result. Therefore, the detection time used was 200 s.

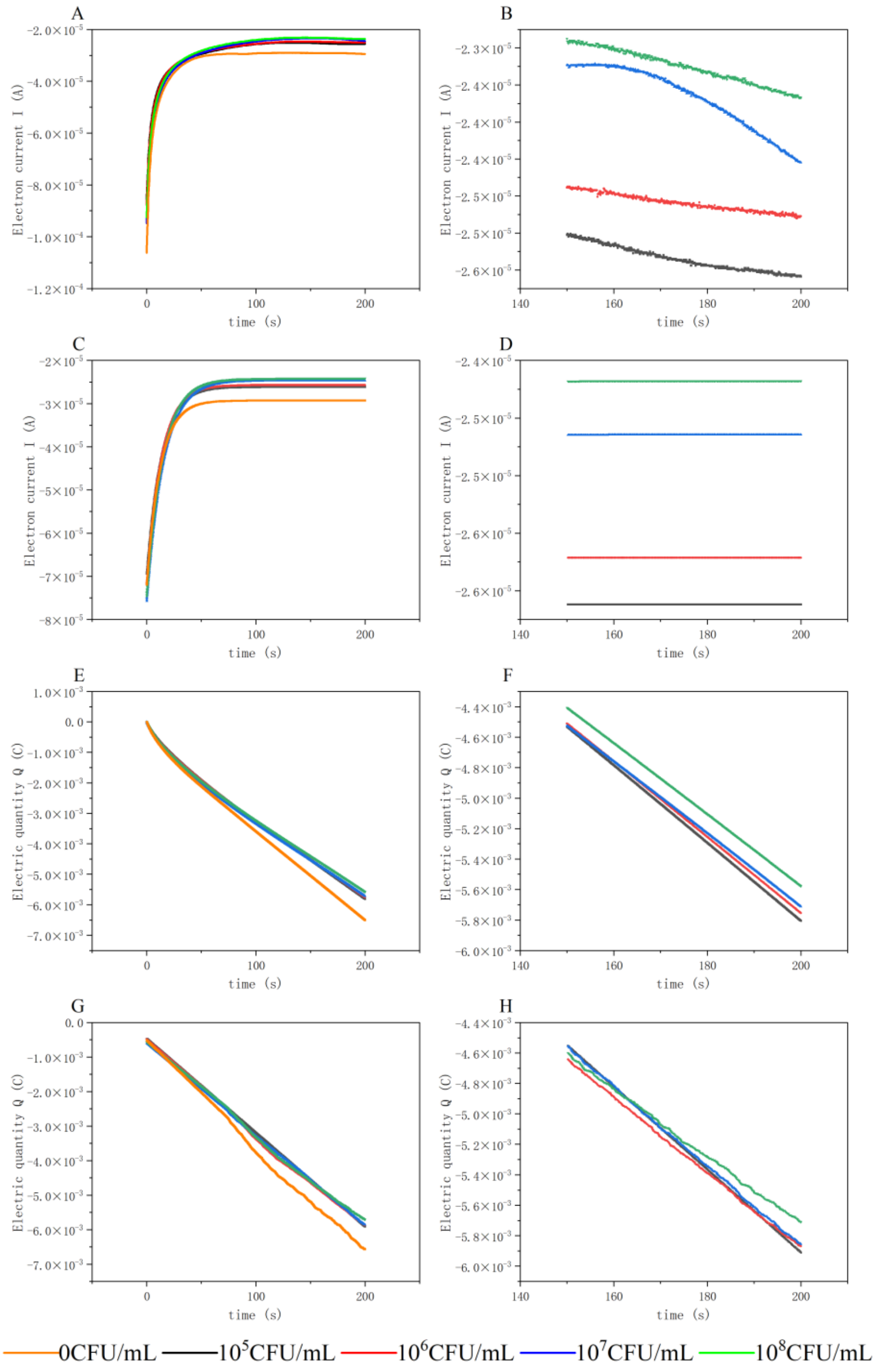

Figure S4. Schematic diagram of curve fitting. A and B, original i-t curve; C and D, fitted i-t curve; E and F, original Q-T curve; G and H, fitted Q-T curve.

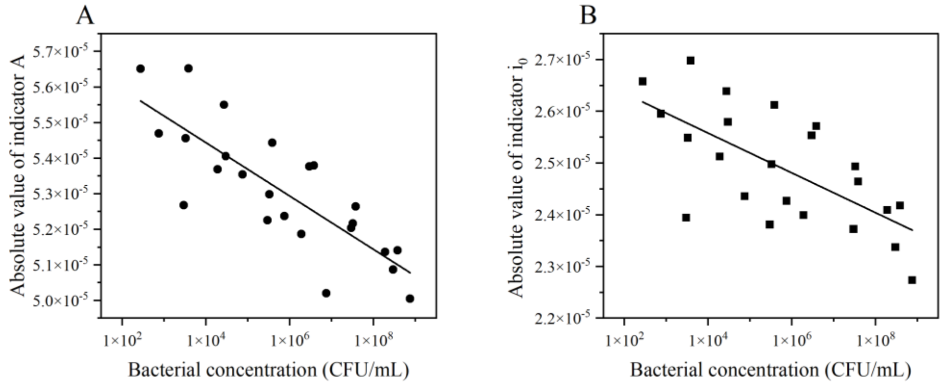

Figure S5. Electrochemical response of the different electrode system to bacterial solution. A, electric quantity index; B, electric current index.

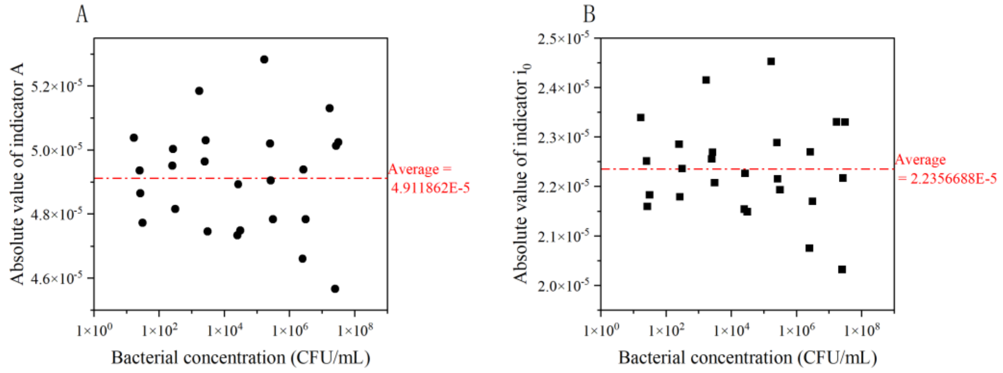

Figure S6. Electrochemical response of *S. aureus*. A, electric quantity index; B, electric current index. Under the same detection method, the electrochemical response of *S. aureus* was completely different from that of *E. coli*, and there was no similar correlation observed in the diagram, so the method of quantify *E. coli* was unsuitable for the analysis of *S. aureus*. This might be because the degree of adsorption of *S. aureus* on the GCE surface had a limited effect on the electrochemical process, but in fact the electrochemical process was still controlled by diffusion.

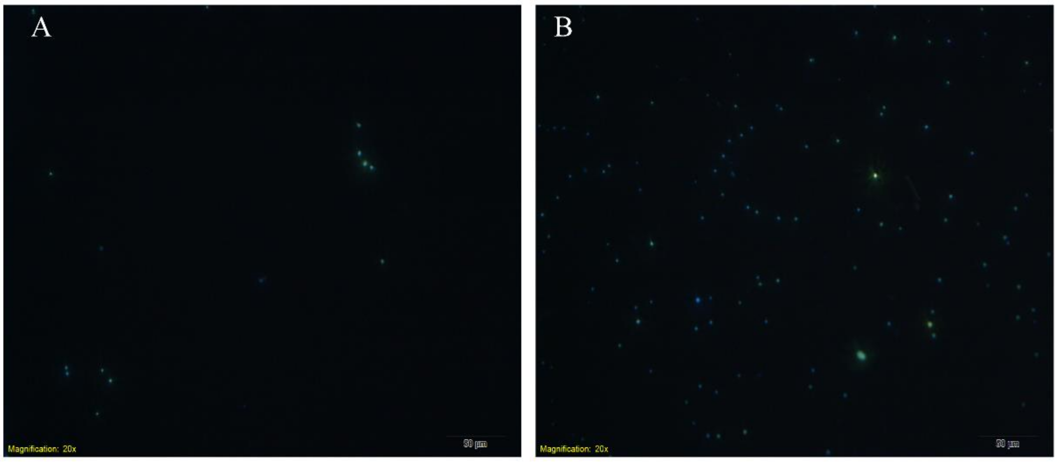

Figure S7. Fluorescence microscopy of the electrode surface after the electrochemical detection of *S. aureus*. A, electrode surface with 10^5 CFU/mL bacteria and B, 10^8 CFU/mL bacteria. When the concentration of *S. aureus* was the same as that of *Escherichia coli*, the amount of *S. aureus* adsorbed on the GCE surface was much lesser than that of *E. coli*. Even if high concentrations of *S. aureus* were electrochemically detected, the amount adsorbed on the GCE surface was still very low. This might be related to some of the characteristics of *S. aureus* mentioned in the main text.

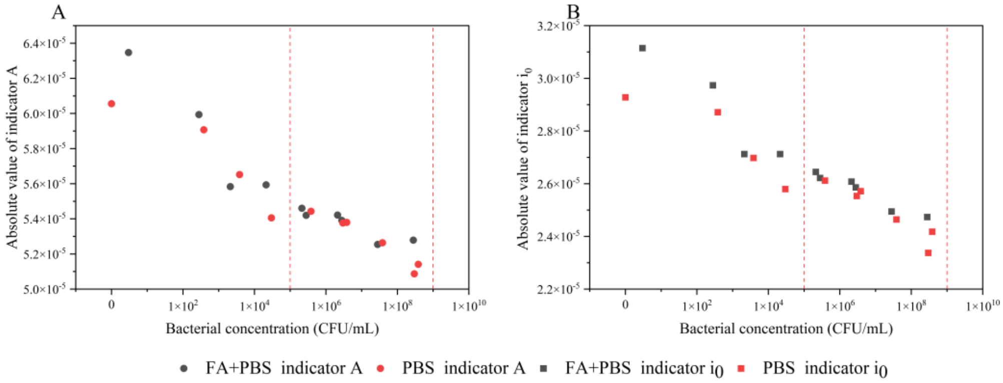

Figure S8. Effect of humic acid on the electrochemical response of *Escherichia coli*. The red mark indicated the control group without humic acid, and the black mark indicated the experimental group containing 10mg/L fulvic acid. A, electric quantity index; B, electric current index.

Table S1. Recoveries of *E. coli* in PBS.

E. coli added (log CFU/mL)	Found (log CFU/mL)	Recovery (%)	RSD (%)
3.86	5.16	133.68	6.54
4.60	5.95	129.35	7.06
5.75	5.42	94.26	3.38
6.60	5.60	84.85	5.74
7.70	6.30	81.82	6.05
8.60	7.44	96.51	4.62

RSD (%) was calculated for the average of 5 teials.

Table S2. Performance comparison of *Escherichia coli* electrochemical biosensors

Electrode modification materials	Labelling	Transduce technique	Range	Detection time	Ref.
--	--	Chronoamperometry	1×10^4-1×10^8 CFU/mL	5min	This study
SiO_2NPs, Abs	--	Cyclic voltammetry	1×10^3-1×10^6 CFU/mL	30min	[1]
AuNPs, PNA, aptamer probes, HRP, aniline	--	Conductance	1×10^3-1×10^8 CFU/mL	3h	[2]
--	MNPs, engineering phages	Linear sweep voltammetry	1×10^5 CFU/mL (LOD)	4h	[3]
--	Au@Pt, NR-rGO@BSA	Cyclic voltammetry	8.9×10^3-8.9×10^9 CFU/mL	--	[4]

References

1. Mga, B, A Cb, A Ig, A Am, C Lb, B Mhd, and A Gh. "Silica Nanoparticles-Assisted Electrochemical Biosensor for the Rapid, Sensitive and Specific Detection of Escherichia Coli." Sensors and Actuators B: Chemical 292 (2019): 314-20.https://doi.org/10.1016/j.snb.2019.03.144
2. Zhang, J., J. Wang, X. Zhang, and F. He. "Rapid Detection of Escherichia Coli Based on 16 S Rdna Nanogap Network Electrochemical Biosensor." Biosensors & Bioelectronics 118 (2018).https://doi.org/10.1016/j.bios.2018.07.041
3. Wang, D., T. Hinkley, J. Chen, J. N. Talbert, and S. R. Nugen. "Phage Based Electrochemical Detection of Escherichia Coli in Drinking Water Using Affinity Reporter Probes." Analyst (2019).https://doi.org/10.1039/C8AN01850B
4. Mo, X., Z. Wu, J. Huang, G. Zhao, and W. Dou. "A Sensitive and Regenerative Electrochemical Immunosensor for Quantitative Detection of Escherichia Coli O157:H7 Based on Stable Polyaniline Coated Screen-Printed Carbon Electrode and Rgo-Nr-Au@Pt." Analytical Methods (2019).https://doi.org/10.1039/C8AY02594K
